# Linking partner violence survivors to supportive services: impact of the M Health Community Network project on healthcare utilization

**DOI:** 10.1186/s12913-019-4313-9

**Published:** 2019-07-12

**Authors:** Cari Jo Clark, Martha Wetzel, Lynette M. Renner, Mary E. Logeais

**Affiliations:** 1 Rollins School of Public Health, Emory University, 1518 Clifton Road, NE, Atlanta, GA 30307 USA; 2 School of Medicine, Emory University, 2015 Uppergate Dr, Atlanta, GA 30322 USA; 3 School of Social Work, University of Minnesota, 1404 Gortner Ave, Peters Hall, St. Paul, MN 55108 USA; 4 School of Medicine, University of Minnesota, 717 Delaware Street SE, Minneapolis, MN 55414 USA

**Keywords:** Partner violence, Screening, Referral, Utilization

## Abstract

**Background:**

Intimate partner violence (IPV) is associated with adverse health effects and increased healthcare utilization. Systems-level interventions have been shown to be effective in identifying and referring survivors but little is known about how these strategies impact future utilization.

The objective of this study is to examine the impact of a systems-level response on healthcare utilization among patients screening positive for IPV from November 2016 to February 2019 in a large multi-specialty outpatient health system in the Midwest.

**Methods:**

Using electronic health record (EHR) data, we identified patients who screened positive for IPV (*N* = 756) and categorized their response as accepted printed material (*N* = 116), accepted direct referrals (*N* = 85), declined both (*N* = 271), or missing (*N* = 255). We used negative binomial models to model post-period utilization as a function of decision group, pre-period utilization, and clinical and demographic factors.

**Results:**

After controlling for demographic characteristics and baseline utilization, the printed materials and direct referral groups had higher utilization rates than those who declined printed materials and direct referral during the post-period for every type of service. However, these differences were only statistically significant for outpatient, behavioral health, and social work services. Specifically, the visit rate for patients receiving printed materials was two times higher (rate ratio: 2.18; 95% CI: 1.21, 3.94) for behavioral health services and three times higher (rate ratio: 3.33; 95% CI: 1.3, 8.52) for social work services compared to those who refused printed material and direct referral. For those opting for a direct referral, the visit rate was two times higher for outpatient services (rate ratio: 1.97; 95% CI: 1.13, 3.42) compared to those who refused.

**Conclusions:**

Patients receiving printed materials or direct referrals had more social work and behavioral health visits, highlighting an important outcome of the protocol. However, higher utilization rates among outpatient services and a trend toward higher utilization of other services, including the emergency department, suggest greater health service utilization is not diminished by the systems level response—at least not within a two-year time frame.

**Electronic supplementary material:**

The online version of this article (10.1186/s12913-019-4313-9) contains supplementary material, which is available to authorized users.

## Background

The top 1% of healthcare utilizers account for 21% of all healthcare spending and are disproportionately more likely to have a history of trauma and complex behavioral needs [1, 2]. One such trauma is intimate partner violence (IPV). IPV is violence or aggression that occurs in a close relationship including current and former spouses or dating partners [3]. Definitions of IPV often include experiences of physical, sexual and emotional forms of violence and stalking [3]. IPV is highly prevalent. Approximately 1 in 4 women and 1 in 7 men in the U.S. have experienced severe physical IPV at some point in their lifetime [4], and IPV is associated with substantial health consequences [5–7]. Healthcare utilization and healthcare costs are higher among individuals who experience IPV [8–16] even after the violence has stopped [12–14, 17].

Healthcare providers who identify and counsel IPV positive patients can reduce victimization [18–21] and positively impact patient health [20, 22, 23]. With the shift toward improved access to preventative services through the Affordable Care Act [24], and the U.S. Preventive Services Task Force recommendation to screen reproductive-aged women and provide appropriate services or referrals (Grade B) [25], healthcare providers have an important role in identifying and referring people who experience IPV to services [26].

There are a growing number of effective healthcare-based interventions for IPV [27–29]. Systems interventions combine IPV screening and referral by healthcare providers, on-site IPV services to respond to victims’ immediate needs, an environment that promotes safe disclosure, and community linkages with strong leadership and oversight [29–32]. IPV-focused, systems-level interventions have been shown to be cost-effective [33] and result in better identification and referral rates compared to more limited screening-only interventions [34]. However, systems interventions are much less frequently employed within healthcare settings compared to more limited IPV screening strategies [35]—which have been shown to be ineffective, alone, at preventing subsequent IPV and may or may not lead to greater referrals to services [36]. Despite the strong linkage between IPV and excess healthcare utilization, the impact of a systems intervention on healthcare utilization is under-researched. Our study begins to fill this gap through a prospective investigation of the impact of the M Health Community Network project on IPV-positive patients’ use of healthcare resources and frequency of missed appointments.

The M Health Community Network project is a collaboration between three groups: (1) academics at the University of Minnesota and Emory University, (2) the Clinics and Surgery Center (CSC), which is a large multi-specialty outpatient health facility jointly administered by the University of Minnesota Physicians and Fairview Health Services, and (3) Domestic Abuse Project (DAP), a therapy, advocacy, and case management service provider. The CSC site is a 342,000 square-foot building, containing 37 adult specialty clinics that serve between 2,000–2,500 patients per day. The CSC includes imaging, diagnostics, laboratory services, an infusion center, clinics, and an ambulatory surgical and procedure center. Approximately 5% of the daily visits to the CSC are for primary care, with the majority of visits in specialty clinics.

The M Health Community Network Project built upon the CSC’s recently formulated multi-disciplinary Behavioral Health Team (BHT) and its organizational tactic to implement a building-wide IPV protocol which routinized identification, response, documentation, and care coordination of adult patients. All adult patients are to be screened for the presence of IPV at least every three months using a four-item validated screening tool [37] that is incorporated into the electronic health record (EHR). Following a positive screen, rooming staff (licensed practice nurses, medical assistants or emergency room technicians who accompany the patient to the exam room and measure vital signs and administer required screens) conduct a five-item lethality assessment and provide resources and/or consult the BHT to provide an immediate in-person response depending on whether a positive screen is associated with a high risk of danger. The BHT social worker or clinician is available to respond to the patient in clinic and provide additional safety and biopsychosocial assessments, as appropriate. The BHT members also provide supportive counseling and referrals to legal, financial, mental health, behavioral health, housing, and other appropriate crisis resources. Based on the patient’s current needs and interest in resources, a direct contact can be made to the community-based agency, DAP. A direct hand-off between a BHT member and the DAP Case Manager supports timely access to crisis resources and referrals. We expect that connecting IPV survivors to behavioral health and social work services would increase their utilization of these services, but that their utilization of other categories of services would be lower than that of survivors who decline services. Further, we expect that connecting survivors to support services would increase their ability to make and keep appointments compared to those not connected to services.

In this study, we report the impact of the M Health Community Network project on healthcare utilization among those screening positive for IPV who accepted referral to supportive services (printed or immediate in-person connection) versus those who did not. Specifically, we test whether patients who chose to receive printed or direct referrals for IPV have less healthcare utilization and fewer missed appointments compared to IPV+ patients who chose not to receive referrals to supportive services over a one-year period. The project received approval from the University of Minnesota (1512 M80888) and Emory University (IRB00094148) Institutional Review Boards. Individual informed consent was waived.

## Methods

### Intervention

**Fig. 1:**
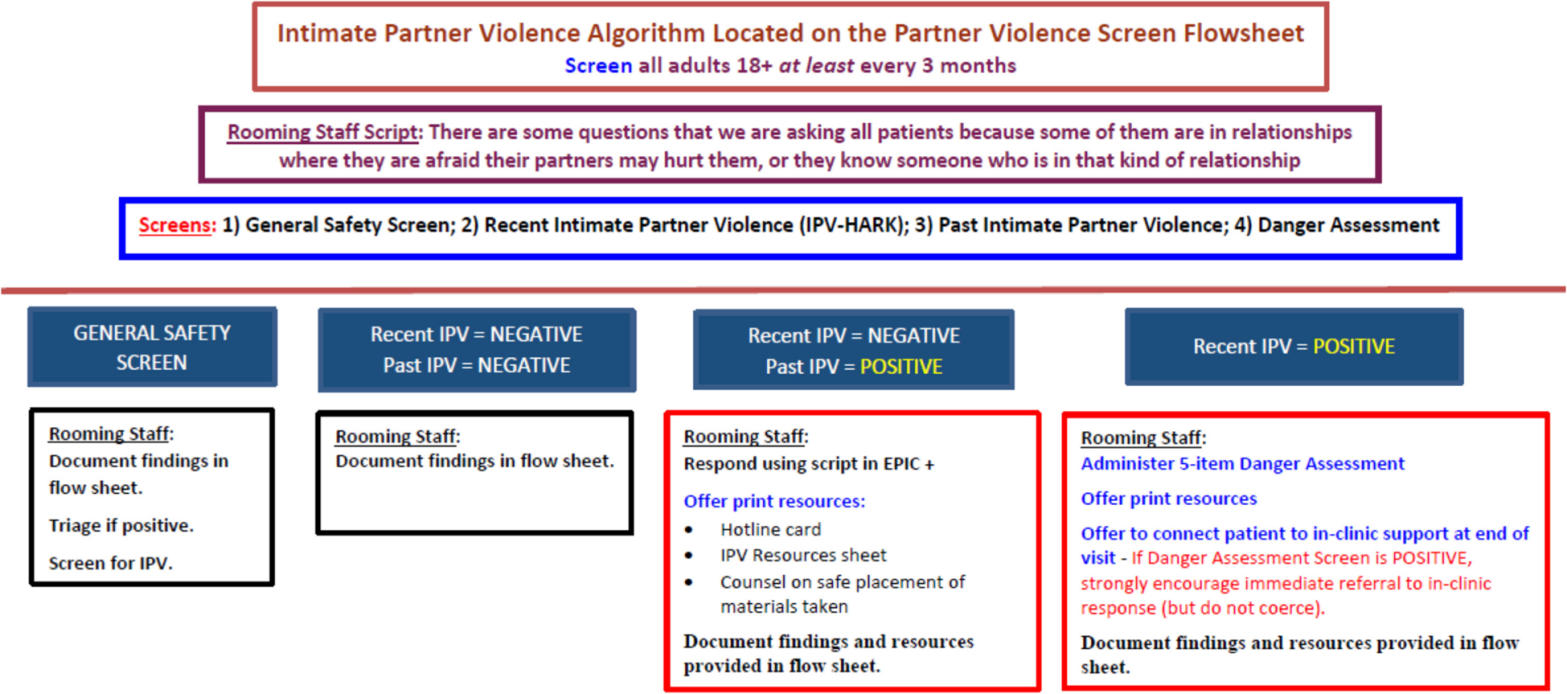
Intimate Partner Violence Protocol

The IPV screening process was embedded in the EHR (EPIC™) and guided by a protocol (Fig. 1). All nursing staff—specifically, rooming staff—received mandatory online and optional in-person training on the protocol. The online and in-person trainings were also made available to all CSC providers, including physicians, behavioral health clinicians, and social workers. According to the protocol, at least every three months, all adult patients were to be screened for IPV using a validated 4-item screening tool (HARK) [37], which is followed by a 5-item Danger Assessment (DA-5) [38] for those who screen positive. The HARK is a 4-item screen to assess past year IPV victimization by a current partner or ex-partner including having been: 1) humiliated or emotionally abused in other ways; 2) afraid; 3) forced to have any kind of sexual activity (word “rape” removed from item with permission); and 4) kicked, hit, slapped or otherwise physically hurt. An additional question was used to screen for prior lifetime IPV. If any item on the HARK instrument was answered affirmatively, the DA-5 was to be administered immediately by the same staff to assess for potential IPV lethality. If the DA-5 score was zero, rooming staff were prompted to provide supportive guidance in the form of scripted text and offer clinic and community-based referral information. If any of the five Danger Assessment items were answered affirmatively, rooming staff were cued to recommend that the patient speak directly with a social work or behavioral health provider during the visit. The rooming staff documented whether the patient accepted or declined direct referral to the social worker/BHT and printed business card sized anonymous contact information for a partnering domestic violence agency. Responses to the screening, danger assessment, and referral items were embedded in a patient flowsheet in the EHR.

### Population

The study population was limited to patients 18 years of age and older who had a positive IPV screen, defined as a ‘yes’ response to any of the four HARK items. From this population, two treatment groups and two comparison groups were created based on the responses to the referral questions. One treatment group (printed material) was comprised of patients who accepted printed materials (i.e., accepted a printout of community resources and/or a confidential domestic violence hotline card). The second treatment group (direct referral) accepted the offer of being directly connected to a BHT or social work staff in the clinic. The first comparison group included patients who did not accept printed or direct referral resources (declined). The second comparison group did not have a documented response to the referral questions (missing).

### Data

Provider information, patient demographics, appointment information, service details, and patient insurance coverage data were extracted from the EHR. Records for patients 18 years and older who had completed at least one response to the HARK were included in the extraction. All records for the included patients from 11/14/2015 through 2/28/2019 were extracted. Analysis of data quality revealed that the patient insurance coverage data was of inadequate quality for inclusion in the study and variables related to type of insurance were removed from the analysis.

Services were categorized into the following mutually-exclusive categories: behavioral health (BH), social work (SW), professional (PR), outpatient (OP), emergency department (ED) and inpatient (IP). These service types were identified per the definitions shown below.

- BH: An in-person or virtual visit in a behavioral health department such as psychiatry or psychology, excluding visits with social workers
- SW: An in-person or virtual visit with a social worker
- PR: An in-person or virtual visit with a professional, except for those classified as MH or SW. Professional visits include primary care visits and visits to specialists, except for those occurring in a hospital setting.
- OP: An in-person hospital-based outpatient clinic visit, except for those classified as MH or SW
- ED: Identified in the EHR as an ED visit
- IP: Inpatient hospital visits

**Fig. 2:**
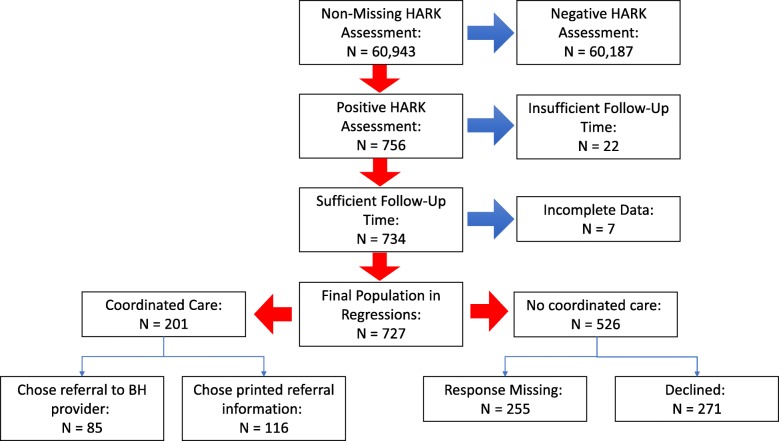
Population Derivation

An index date demarcating the boundary between the pre and post periods was assigned to all patients. For the treatment populations, the index date was defined as the earliest visit at which they accepted printed material or a direct referral. By necessity, the index date for the comparison populations was defined using a different algorithm. For the primary analysis, the index date for the comparison groups was defined as the earliest date at which they had a positive HARK screening. As a sensitivity analysis, the index date was randomly selected from the patients’ HARK positive screening dates. In all cases, patients with index dates falling within one month of 2/28/2019 were excluded due to insufficient follow-up time. Figure 2 presents the population derivation.

### Data analysis

In order to calculate visits per year, baseline patient years were calculated by determining the number of months between the start of the measurement period (11/14/2015), which is approximately one year prior to the start of the intervention (11/16/2016), and the index date. Patient months were then converted to patient years. We did not have sufficient data to determine when individuals first became patients within the CSC health system; however, we determined that there was no difference in the average number of days between the start of the measurement period and IPV diagnosis among the four compared groups. For the treatment period, patient years were calculated based on the time from the index date through the end of the measurement period, 2/28/2019.

One additional outcome, missed appointments, was included in the analysis. Missed appointments were modeled as the number of missed appointments per patient year, adjusted for the number of appointments made. Healthcare utilization data were collected from all visits occurring within the wider health system, and not just in the CSC. The data were obtained via a clinical data repository containing information from over 2.5 million patients seen by one of the health practice partners at practice sites including 8 hospitals and over 40 clinics enabling the assessment of visits across the spectrum (primary, secondary, tertiary and quaternary). Informatics specialists managed data extraction and basic data management on behalf of study investigators. The data requisition form is available upon request.

Bivariate and regression analyses were performed for all outcomes, excluding IP due to the small number of IP visits. All outcomes showed evidence of over-dispersion and were modeled as negative binomials with logged post-period patient years included as an offset. The focal predictor was the coordinated care group (i.e., printed materials, direct referral, declined, or missing). The general form of the equations was number of visits as a function of coordinated care group and controls with the patient year offset. Control variables included age, sex, race, revised Charlson Comorbidity Index [39], DA-5 score, type of IPV, received primary care within the system, and baseline period utilization rates. The missed appointment rate model also included the total number of appointments made during the post-period. All analyses were performed using SAS software version 9.4.

## Results

A total of 727 patients met the inclusion criteria. Responses to the printed materials and/or direct referral were entered for approximately 65% of patients. Twenty-eight percent of patients who screened positive for IPV accepted either printed materials or a direct referral during the measurement period. There were clear differences between the groups in HARK responses and DA-5 scores. The patients in the printed material and direct referral groups reported being afraid of their partners at the rates of 61.2 and 63.5% respectively versus approximately 45% of both the comparison groups. Additionally, patients in the direct referral group were the most likely to report having been sexually abused by their partner. Both the printed material and direct referral groups scored higher on the DA-5 compared to both comparison groups and the printed material group had the highest utilization rate for behavioral health services. Descriptive statistics are provided in Table 1.

**Table 1:** Descriptive Statistics

Variable	Level	N	Missing N = 255	Declined N = 271	Printed Materials N = 116	Direct Referral N = 85	*P*-Value
Sex	Female	727	205 (80.39%)	208 (76.75%)	97 (83.62%)	58 (68.24%)	**0.046**
	Male		50 (19.61%)	63 (23.25%)	19 (16.38%)	27 (31.76%)	
Race: White		727	180 (70.59%)	215 (79.34%)	82 (70.69%)	52 (61.18%)	**0.006**
Race: Black		727	27 (10.59%)	27 (9.96%)	15 (12.93%)	19 (22.35%)	**0.017**
Race: Other		727	16 (6.27%)	*	*	*	
Race: Missing		727	32 (12.55%)	20 (7.38%)	14 (12.07%)	12 (14.12%)	0.152
Ethnicity	Hispanic or Latino	673	9 (3.78%)	*	*	*	
	Not Hispanic or Latino		229 (96.22%)	*	*	*	
HARK: Humiliate		727	211 (82.75%)	214 (78.97%)	94 (81.03%)	74 (87.06%)	0.365
HARK: Fear Item		727	118 (46.27%)	124 (45.76%)	71 (61.21%)	54 (63.53%)	**0.002**
HARK: Sexual Abuse Item		727	42 (16.47%)	47 (17.34%)	18 (15.52%)	29 (34.12%)	**0.002**
HARK: Physical Abuse Item		727	51 (20.00%)	66 (24.35%)	44 (37.93%)	37 (43.53%)	**<.001**
PCP Visit Within the Health System		727	136 (53.33%)	96 (35.42%)	48 (41.38%)	32 (37.65%)	**<.001**
Age		727	43 (32, 59)	42 (31, 56)	35 (24.5, 47)	43 (28, 53)	**<.001**
Danger Assessment Score		586	0 (0, 1)	0 (0, 1)	1 (0, 1)	1 (0, 2)	**<.001**
CCI		727	1 (0, 3)	1 (0, 3)	0 (0, 1)	0 (0, 2)	**<.001**
Days Until Diagnosis		727	435 (168, 677)	442 (231, 725)	493.5 (187, 808.5)	522 (267, 744)	0.221
Months of Follow-Up		727	17 (9, 22)	18 (11, 23)	11 (7.5, 18)	13 (9, 21)	**<.001**
Missed Appointments Rate in Baseline		727	0.38 (0.00, 1.85)	0.67 (0.00, 2.25)	0.86 (0.00, 2.67)	0.92 (0.00, 2.53)	**0.047**
Behavioral Heath Visit Rate in Baseline		727	0.00 (0.00, 0.48)	0.33 (0.00, 4.00)	2.70 (0.57, 10.34)	0.00 (0.00, 4.00)	**<.001**
Social Work Visit Rate in Baseline		727	0.00 (0.00, 0.00)	0.00 (0.00, 0.00)	0.00 (0.00, 0.17)	0.00 (0.00, 0.00)	0.612
Professional Visit Rate in Baseline		727	4.00 (0.71, 11.29)	5.00 (1.00, 13.33)	3.13 (0.86, 9.09)	3.56 (0.43, 10.36)	0.110
Outpatient Visit Rate in Baseline		727	1.29 (0.00, 5.54)	0.60 (0.00, 3.00)	0.00 (0.00, 0.98)	0.35 (0.00, 2.77)	**<.001**
Emergency Department Visit Rate in Baseline		727	0.00 (0.00, 0.92)	0.00 (0.00, 1.24)	0.00 (0.00, 0.80)	0.34 (0.00, 0.86)	0.147
Inpatient Stay Rate in Baseline		727	0.00 (0.00, 0.00)	0.00 (0.00, 0.00)	0.00 (0.00, 0.00)	0.00 (0.00, 0.00)	0.329

Note: Days until diagnosis was calculated as the number of days between first document visit during the measurement period and date of first positive HARK screening. Months of follow-up was calculated as months between index date and end of the measurement period. All baseline visit rates represent the median visits per year, with the denominator calculated as time in years between beginning of measurement period and index date

*Indicates results omitted due to small cell sizes

PCP: Primary care practitioner

CCI: Charlson Comorbidity Index

**Fig. 3:**
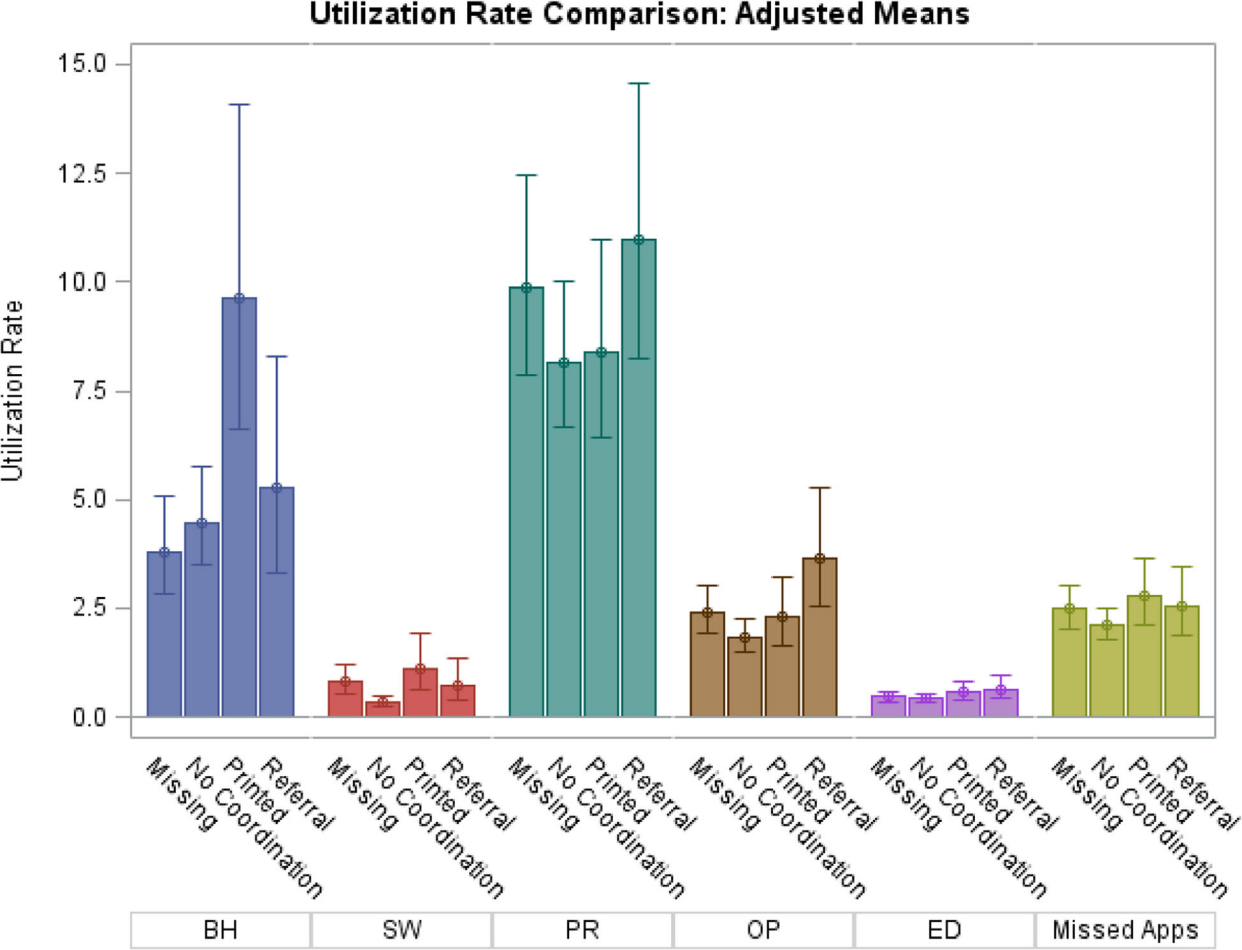
Average Adjusted Utilization Rates

Least squared means and utilization rate ratios from the regression analyses are shown in Fig. 3 and Table 2, respectively. After controlling for demographic characteristics and baseline utilization, the printed materials and direct referral groups had higher utilization rates than those who declined printed materials and direct referral during the post-period for every type of service. However, these differences were only statistically significant for outpatient, behavioral health, and social work services. Specifically, the visit rate for patients receiving printed materials was two times higher (rate ratio: 2.18; 95% CI: 1.21, 3.94) for behavioral health services and three times higher (rate ratio: 3.33; 95% CI: 1.3, 8.52) for social work services compared to those who refused printed material and direct referral. For those opting for a direct referral, the visit rate was two times higher for outpatient services (rate ratio: 1.97; 95% CI: 1.13, 3.42) compared to those who refused. The pairwise comparisons also showed that the missing group’s visit rate for social work was 2.5 times that of the declined group (rate ratio: 2.48, 95% CI: 1.11–5.51). The missing group had lower behavioral utilization 0.39 (0.20, 0.75) compared to those receiving printed materials. The sensitivity analysis results were nearly identical to the primary analysis results and are included in Additional file 1: Table S1.

**Table 2:** Adjusted Utilization Rate Ratios

Service	Adjusted Rate Ratios (95% CI)						Overall *P*-Value
	Missing to Printed	Missing to Direct Referral	Missing to Declined	Printed to Direct Referral	Printed to Declined	Direct Referral to Declined	
Behavioral Health	0.39 (0.20, 0.75)*	0.70 (0.33, 1.49)	0.85 (0.49, 1.45)	1.80 (0.84, 3.87)	2.18 (1.21, 3.94)*	1.21 (0.62, 2.39)	**0.001**
Social Work	0.74 (0.29, 1.93)	1.10 (0.39, 3.10)	2.48 (1.11, 5.51)*	1.48 (0.49, 4.44)	3.33 (1.3, 8.52)*	2.26 (0.82, 6.23)	**0.003**
Professional	1.15 (0.79, 1.67)	0.88 (0.59, 1.31)	1.20 (0.89, 1.61)	0.77 (0.50, 1.18)	1.05 (0.74, 1.47)	1.36 (0.94, 1.98)	0.123
Outpatient	1.07 (0.61, 1.88)	0.67 (0.38, 1.2)	1.32 (0.87, 2.01)	0.63 (0.33, 1.20)	1.23 (0.73, 2.08)	1.97 (1.13, 3.42)*	**0.013**
Emergency Department	0.82 (0.44, 1.54)	0.72 (0.37, 1.39)	1.09 (0.67, 1.77)	0.87 (0.43, 1.79)	1.33 (0.74, 2.37)	1.52 (0.81, 2.86)	0.314
Missed Appointments	0.89 (0.56, 1.42)	0.98 (0.59, 1.60)	1.18 (0.82, 1.69)	1.09 (0.65, 1.83)	1.32 (0.87, 1.99)	1.21 (0.76, 1.91)	0.306

*Indicates pairwise comparison significant at the *p* < 0.05 level

Adjusted for gender, race, HARK items, age, Danger Score, CCI, and baseline rates for missed appointments, behavioral health, social work, professional, outpatient, emergency department, and inpatient visits

## Discussion

In this study, we examined the impact of a systems-level response on healthcare utilization among patients who screen positive for IPV. In alignment with best practices, we implemented an intervention that included a standardized process to assess IPV and offer referrals to supportive services as appropriate in a large multi-specialty health system. As anticipated, patients accepting printed material or a direct referral to the BHT or social worker had higher social work and behavioral health utilization in the post-period than patients declining services, although only statistically significant for the printed referral group. This provides evidence that the group choosing some form of referral is receiving follow-up behavioral health and/or social work support. However, higher utilization rates among outpatient services, also suggests an underlying phenomenon of greater health service utilization that is not diminished by the referral system—at least not within the time frame being examined.

A number of explanations are likely. The two-year follow-up time may be insufficient to make significant impacts on healthcare utilization among patients whose lives and medical profile are complex. It may be that IPV-aware care leads to appropriately higher utilization initially, such that patients are engaging with the healthcare system in a meaningful way that supports management of their medical comorbidities as a direct result of the support they have received. Further analyses with longer follow-up time may be needed to identify trends towards diminishing unnecessary healthcare utilization, especially emergency department use and fewer missed appointments which were elevated in those receiving printed materials and direct referrals despite attempts to connect them to supportive services.

It may also be that the supportive care provided was not intensive enough. Our M Health Community Network project emphasizes warm hand-offs to support service providers and approximately 40% of the patients accepting resources selected the direct referral over printed materials; however, it is still possible that the support services were insufficient. We were also unable to document what services were ultimately provided versus those that were offered. The items that we incorporated into the EHR for this project were a meaningful step forward in systematic documentation; however, the use of standard EHR ‘smart phrases’ would yield more specificity on subsequent support services provided. Further, 35% of individuals with a positive screen had missing data in the EHR for both the referral and printed questions. Patients with missing referral data had greater social work utilization than those declining referral but less behavioral health utilization than those receiving printed referrals. This suggests that these patients had some need for social work services, but given the lack of information about referrals for IPV, it is difficult to ascertain what occurred in those visits. The presence of missing data is unfortunate but reflects the reality of medical practice and the use of EHR data to assess intervention impact.

In addition to testing the impact of the intervention on healthcare utilization, our study is novel in that it assesses the relationship between IPV and health utilization via a built-in mechanism for a two-tiered referral system, generating direct referrals for IPV survivors with high levels of acuity on a danger assessment. It also compares demographic and health utilization patterns among two different referral strategies in one study. Although previous researchers have examined past victimization in relation to current victimization and cumulative exposure [40], to our knowledge, none have examined IPV acuity and health resource utilization. Greater utilization among patients with the most potentially-lethal IPV aligns well with prior researchers who found higher utilization among patients who experience IPV compared to patients not reporting IPV [8–17, 41, 42].

## Limitations

Limitations of this study include the use of relatively small population size, particularly in the intervention groups, although data stem from a database which is part of a large university / private health care partnership offering greater generalizability of the findings than if restricted to one healthcare provider. The partnership in care and data sharing means that coverage is extensive and includes patient services from primary to quaternary care. The requirement that the patient have a primary care visit within the system suggests that fewer visits are missed that may have occurred outside the system, although some missed healthcare utilization is likely. The measurement period was limited to just over 2 years and relied on data that were feasible to collect regarding the response provided to the patient, but ideally more nuanced information about what follow-up services entailed would help to discern the intensity of the care coordination and the appropriateness of subsequent health care utilization. Additionally, due to the large study setting size and heterogeneity among clinic sites, it was difficult to control or measure differences in the execution of the protocol. Variations in staffing, clinic processes and protocol adherence may have negatively influenced the number of patients who received referrals, though these are relatively common barriers in large health system quality improvement initiatives.

## Conclusion

Healthcare providers who screen for IPV and provide patients with referrals to appropriate services can positively impact patient safety and well-being. However, screening rates among health care providers are notoriously low, with an estimated 2–50% of medical professionals performing routine screening for female patients [43]. Our project addressed this challenge by implementing routine IPV screening and an integrated response within a large clinic system. Our findings demonstrate that the prevention and response protocol seems to be working to identify, refer and connect those with higher lethality scores to helping resources. Further research, particularly over a longer time frame is needed to ascertain if the identification and referral protocol is resulting in enhanced patient safety and wellbeing, highlighting the need to invest in the long term when working to ameliorate complex social-health issues such as IPV.

## Additional file

Additional file 1: **Table S1**. Sensitivity Analysis Results Adjusted Utilization Rate Ratios Adjusted utilization rate ratios for sensitivity analyses. (DOCX 13 kb)

## Data Availability

Access to the clinical data repository and informatics support is restricted to researchers at the University of Minnesota, Fairview, and University of Minnesota Physicians, and is provided through the Best Practice Integrated Informatics Core (BPIC). The algorithm that was used to generate the dataset and the statistical programs used to compute the findings are available upon reasonable request from the corresponding author.

## Abbreviations

BH: Behavioral health
BHT: Behavioral health team
CCI: Charlson Comorbidity Index
CSC: Clinics and Surgery Center
DA: Danger Assessment
DAP: Domestic Abuse Project
ED: Emergency department
EHR: Electronic health record
HARK: Acronym for the intimate partner violence screen
IP: Inpatient
IPV: Intimate partner violence
OP: Outpatient
PCP: Primary care provider
PR: Professional
SW: Social work
