# Supplementary material for: Linking partner violence survivors to supportive services: impact of the M Health Community Network project on healthcare utilization
Source: BMC Health Serv Res. 2019 Jul 12;19:479. doi: 10.1186/s12913-019-4313-9 (PMC6624981; doi:10.1186/s12913-019-4313-9)
Supplement: Supplementary file 1 — Table S1. Sensitivity Analysis Results Adjusted Utilization Rate Ratios Adjusted utilization rate ratios for sensitivity analyses. (DOCX 13 kb) [file 12913_2019_4313_MOESM1_ESM.docx]

Supplemental Table 1: Sensitivity Analysis Results – Adjusted Utilization Rate Ratios

| Service | Adjusted Rate Ratios (95% CI) | | | | | | Overall  P-Value | |
| --- | --- | --- | --- | --- | --- | --- | --- | --- |
|  | Missing to Printed | Missing to Referral | Missing to No Coordination | Printed to Referral | Printed to No Coordination | Referral to No Coordination |  |  |
| Behavioral Health | 0.38 (0.19, 0.73)* | 0.67 (0.31, 1.44) | 0.86 (0.5, 1.47) | 1.79 (0.83, 3.84) | 2.29 (1.27, 4.12)* | 1.28 (0.65, 2.51) | <0.001 | |
| Social Work | 0.75 (0.29, 1.94) | 1.11 (0.4, 3.14) | 2.39 (1.08, 5.28)* | 1.49 (0.5, 4.46) | 3.19 (1.26, 8.08)* | 2.14 (0.78, 5.87) | 0.005 | |
| Professional | 1.13 (0.78, 1.65) | 0.87 (0.58, 1.3) | 1.19 (0.88, 1.6) | 0.77 (0.5, 1.18) | 1.05 (0.75, 1.47) | 1.37 (0.94, 1.99) | 0.130 | |
| Outpatient | 1.01 (0.58, 1.78) | 0.64 (0.36, 1.15) | 1.28 (0.84, 1.95) | 0.63 (0.33, 1.22) | 1.26 (0.74, 2.15) | 2 (1.15, 3.49)* | 0.013 | |
| ED | 0.78 (0.42, 1.48) | 0.68 (0.35, 1.32) | 1.08 (0.66, 1.76) | 0.87 (0.42, 1.78) | 1.37 (0.76, 2.46) | 1.58 (0.84, 2.97) | 0.227 | |
| Missed Appointments | 0.9 (0.56, 1.43) | 0.98 (0.6, 1.62) | 1.18 (0.82, 1.69) | 1.1 (0.66, 1.83) | 1.31 (0.87, 1.98) | 1.2 (0.76, 1.89) | 0.320 | |
| **Indicates pairwise comparison significant at the p < 0.05 level.* | | | | | | | |  |
|  | | | | | | | | |
